# Advancing rheumatology with natural language processing: insights and prospects from a systematic review

**DOI:** 10.1093/rap/rkae120

**Published:** 2024-09-19

**Authors:** Mahmud Omar, Mohammad E Naffaa, Benjamin S Glicksberg, Hagar Reuveni, Girish N Nadkarni, Eyal Klang

**Affiliations:** Faculty of Medicine, Tel-Aviv University, Tel Aviv, Israel; Rheumatology Unit, Galilee Medical Center, Nahariya, Israel; Charles Bronfman Institute for Personalized Medicine, Icahn School of Medicine at Mount Sinai, New York, New York, USA; Division of Data-Driven and Digital Medicine (D3M), Icahn School of Medicine at Mount Sinai, New York, New York, USA; Division of Diagnostic Imaging, Sheba Medical Center, Affiliated to Tel-Aviv University, Ramat Gan, Israel; Charles Bronfman Institute for Personalized Medicine, Icahn School of Medicine at Mount Sinai, New York, New York, USA; Division of Data-Driven and Digital Medicine (D3M), Icahn School of Medicine at Mount Sinai, New York, New York, USA; Charles Bronfman Institute for Personalized Medicine, Icahn School of Medicine at Mount Sinai, New York, New York, USA; Division of Data-Driven and Digital Medicine (D3M), Icahn School of Medicine at Mount Sinai, New York, New York, USA

**Keywords:** large language models (LLMs), natural language processing (NLP), rheumatology, artificial intelligence (AI), disease detection

## Abstract

**Objectives:**

Natural language processing (NLP) and large language models (LLMs) have emerged as powerful tools in healthcare, offering advanced methods for analysing unstructured clinical texts. This systematic review aims to evaluate the current applications of NLP and LLMs in rheumatology, focusing on their potential to improve disease detection, diagnosis and patient management.

**Methods:**

We screened seven databases. We included original research articles that evaluated the performance of NLP models in rheumatology. Data extraction and risk of bias assessment were performed independently by two reviewers, following Preferred Reporting Items for Systematic Reviews and Meta-Analyses guidelines. The Quality Assessment Tool for Observational Cohort and Cross-Sectional Studies was used to evaluate the risk of bias.

**Results:**

Of 1491 articles initially identified, 35 studies met the inclusion criteria. These studies utilized various data types, including electronic medical records and clinical notes, and employed models like Bidirectional Encoder Representations from Transformers and Generative Pre-trained Transformers. High accuracy was observed in detecting conditions such as RA, SpAs and gout. The use of NLP also showed promise in managing diseases and predicting flares.

**Conclusion:**

NLP showed significant potential in enhancing rheumatology by improving diagnostic accuracy and personalizing patient care. While applications in detecting diseases like RA and gout are well developed, further research is needed to extend these technologies to rarer and more complex clinical conditions. Overcoming current limitations through targeted research is essential for fully realizing NLP’s potential in clinical practice.

Key messages

- NLP models exhibit high accuracy in detecting conditions such as rheumatoid arthritis, spondyloarthropathies and gout from clinical texts.
- LLMs show potential in addressing patient inquiries and enhancing education in rheumatology with high precision.
- Further research is needed on NLP applications in rare rheumatic diseases and personalized treatment planning.

## Introduction

Healthcare is rapidly evolving, driven by significant artificial intelligence (AI) advancements [1]. Among these, natural language processing (NLP) and especially large language models (LLMs) have emerged as transformative technologies [2, 3].

NLP and LLMs introduce methods for analysing unstructured clinical texts [2, 3]. These technologies can extract information from electronic health records (EHRs), improving patient care, research and administrative work [4–6]. The ability of LLMs to understand context and interpret complex medical terminology makes them valuable tools for clinicians and researchers [7, 8].

Rheumatology, a field characterized by diverse disorders, can benefit from these advancements [9]. Rheumatological conditions often involve multiple organ systems and present with overlapping symptoms, making accurate diagnosis challenging [10]. NLP offers the potential to extract relevant clinical data, enhance disease classification and support decision-making [4, 5, 11, 12].

Despite its promise, NLP adoption for rheumatology has been relatively slow [13, 14]. This lag is due in part to the complexity of the field and the need for highly accurate tools [13]. However, recent studies have demonstrated the feasibility of NLP in various aspects of rheumatology, including disease detection, patient management and education [5, 12, 15]. These studies highlight the potential for NLP to address some of the most pressing challenges in the field.

Our review aims to provide insights into the current state of NLP research in rheumatology and identify areas for future clinical application.

## Materials and methods

### Registration and protocol

This systematic literature review was registered with the International Prospective Register of Systematic Reviews (PROSPERO) under the registration code CRD42024509490 [16]. Our methodology adhered to the Preferred Reporting Items for Systematic Reviews and Meta-Analyses (PRISMA) guidelines [17].

### Search strategy

We searched seven databases: PubMed, Embase, Web of Science, Scopus, Cochrane Library, IEEE Xplore and OVID-MEDLINE. The search covered studies published between 1 January 2002 and April 2024. The start date was chosen because it marks the announcement of the neural probabilistic language model, foundational for the application of NLP in medicine [18, 19]. Our focus was on the outcomes of integrating NLP and LLMs in rheumatology. We used keywords like ‘natural language processing,’ ‘NLP,’ ‘large language models’ and ‘LLMs’, along with specific model names and rheumatological terms like ‘GPT,’ ‘BERT,’ ‘rheumatoid arthritis’ and ‘gout’. We designed Boolean search strings tailored to each database. To maximize coverage, we supplemented our search with a manual reference screening of included studies and targeted searches on Google Scholar and medrxiv. Details of the specific Boolean strings used are provided in the supplementary materials (available at *Rheumatology Advances in Practice* online).

### Study screening and selection

We included articles that directly evaluated the performance of NLP models in rheumatology applications and provided data about the performance, either qualitative or quantitative.

Our review encompasses original research articles and full conference articles [20]. The exclusion criteria were confined to review articles, case reports, commentaries, protocol studies, editorials and non-English publications, in addition to articles that did not directly evaluate the model performance.

For the initial screening, we used the Rayyan web application [21]. The initial screening and study selection, which were conducted according to predefined criteria, were independently performed by two reviewers (M.O. and E.K.). Discrepancies were resolved through discussion.

### Data extraction

Data extraction was conducted by two researchers (M.O. and E.K.) using a standardized form to ensure consistent and accurate data capture. This included details such as author, publication year, sample size, data type, task type, disease interest, model used, results, performance metrics, conclusions and limitations. Any discrepancies in data extraction were resolved through discussion and a third reviewer was consulted when necessary.

### Risk of bias assessment

To evaluate the quality and robustness of the methodologies in the included studies, the Quality Assessment Tool for Observational Cohort and Cross-Sectional Studies tool was used [22].

## Results

**Figure 1.**
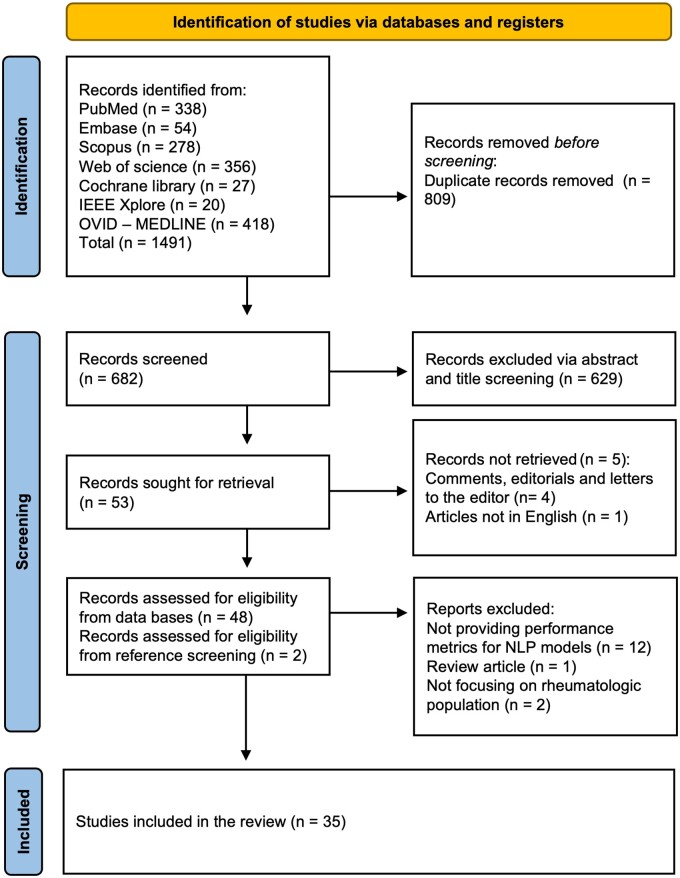
PRISMA flow chart

A total of 1491 articles were identified through the initial screening. After removing 809 duplicates, 682 articles remained for further evaluation. Title and abstract screening excluded 629 articles, leaving 53 articles for full-text review. From these, 34 studies met all inclusion criteria. By using reference checking and snowballing techniques, one additional study was identified, resulting in a final tally of 35 studies [5, 6, 12, 23–54]. A PRISMA flow chart visually represents the screening process in Fig. 1.

### Overview of the included studies

We included 35 studies [5, 6, 12, 23–54], spanning from 2010 to 2024. Analysed sample sizes ranged from a few hundred to >2 million patients. The studies utilized various data types, including electronic medical records (EMRs), structured and unstructured electronic health records (EHRs) data, clinical notes and radiology reports.

Employed models included advanced NLP techniques, ensemble models and specific LLM architectures like Bidirectional Encoder Representations from Transformers (BERT) and Generative Pre-trained Transformer (GPT).

We categorized the applications into two main groups: patient care and detection and diagnosis (Table 1). Eleven studies focused on patient care, evaluating models in answering patient questions, predicting flares, classifying disease severity and managing diseases. Twenty-four studies focused on detection and diagnosis, identifying diseases or flares from data and making diagnoses, such as identifying gout flares, detecting pain levels in OA and diagnosing RA.

**Table 1.** Summary of the included studies

Author, year [ref]	Data type and sample size	Model	Summary of most important results
Chen *et al.*, 2023 [48]	EMR, data from >2 million patients	NLP (NER, POS)	Synonym-based pain-level detection tool accurately identified patients with moderate–severe pain due to OA
Saini *et al.*, 2023 [29]	X-ray image reports, structured EHR data, 4508 patients	CNN, YOLO v4, Transformer, BERT	High performance in predicting knee OA severity and generating reports with AUROCs from 0.897 to 0.9582.
Benavent *et al.*, 2024 [5]	Unstructured EHR data, 4337 patients	NLP-based system	High precision, recall and F1 scores (>0.80) for detecting clinical entities related to SpA
Li *et al.*, 2022 [46]	EMRs, 1600 clinical notes	BERT	Improved NER in clinical notes with an F1 score of 0.936
Krusche *et al.*, 2024 [31]	Patient vignettes, 20 different real-world patient vignettes	GPT-4	Comparable diagnostic accuracy to rheumatologists for IRDs, with top diagnosis accuracy of 35%
Madrid-García *et al.*, 2023 [39]	Exam questions from Spanish access exam, 145 questions	GPT-4	GPT-4 showed 93.71% accuracy in answering rheumatology questions
Irfan and Yaqoob 2023 [23]	Database of peer-reviewed articles and clinical guidelines	GPT-4	Provided insights into SS, highlighting key characteristics and management details
Nelson *et al.*, 2015 [49]	Medical text infusion notes, 115 patients, 2029 infliximab infusions	Custom rule-based NLP software	Improved sensitivity (0.858) and PPV (0.976) for identifying infliximab infusion dates and doses
Liu *et al.*, 2023 [25]	Chinese EMRs, 1986 CEMRs	MC-BERT-BiLSTM-CRF, MC-BERT + FFNN	Achieved F1 scores of 92.96% for NER and 95.29% for relation extraction
Humbert-Droz *et al.*, 2023 [30]	Clinical notes from the RISE registry, 854 628 patients	NLP pipeline (Spacy)	Sensitivity, PPV and F1 scores of 95%, 87% and 91%, respectively, for RA outcome measures extraction
Benavent *et al.*, 2023 [6]	Free-text and structured clinical information, 758 patients	EHRead technology	High performance in identifying clinical variables for axSpA and PsA, precision of 0.798 and recall of 0.735 for PsA
VanSchaik *et al.*, 2023 [53]	PubMed abstracts, 2350 abstracts	ELECTRA-based model	Extracted causal relationships with an F1 score of 0.91
Walsh *et al.*, 2020 [40]	Clinical notes, structured EHR data, 600 patients	NLP algorithms with random forest	AUROC of 0.96 for full algorithm in identifying axSpA
Yoshida *et al.*, 2024 [42]	EHR notes and Medicare claims data, 500 patients	LASSO	Combined model showed an AUROC of 0.731 for identifying gout flares
Li *et al.*, 2023 [52]	FAQ-based question-answering pairs, 176 questions	BERT, RoBERTa, ALBERT, MacBERT	Achieved top-1 precision of 0.551 and MRR of 0.660 in an RA question-answering system
Ye *et al.*, 2024 [33]	Patient-generated rheumatology questions, 17 patients	GPT-4	Patients rated AI responses similarly to physician responses; rheumatologists rated AI lower in comprehensiveness
Coskun *et al.*, 2024 [23]	Questions on methotrexate use, 23 questions	GPT-4, GPT-3.5, BARD	GPT-4 achieved 100% accuracy in providing information on methotrexate use
Liao *et al.*, 2010 [36]	Narrative and codified EMR data, 29 432 subjects	HITEx system	Improved RA classification accuracy with a PPV of 94% using narrative and codified data
Lin *et al.*, 2015 [24]	Structured and unstructured EHR data, 5903 patients	Apache cTAKES, ML	PPV of 0.756, sensitivity of 0.919 and F1 score of 0.829 for identifying methotrexate-induced liver toxicity
Wang *et al.*, 2017 [32]	Spontaneous reports, EMRs, 138 000 patients	MedEx, UMLS, MedDRA PT codes	Detected 152 signals for biologics and 147 for DMARDs from clinical notes
Uz and Umay, 2023 [34]	Structured EHR data and internet search data	ChatGPT	Reliability scores ranged from 4 to 7, with the highest for OA (5.62); usefulness scores highest for AS (5.87)
Luedders *et al.*, 2023 [37]	Chest CT reports, 650 patients	Automated regular expressions	Improved PPV to 94.6% for RA-ILD identification
Osborne *et al.*, 2024 [41]	Chief complaint text from emergency department, 8037 CCs	Rule-based, BERT-based algorithm	BERT-GF achieved an F1 score of 0.57 for detecting gout flares
Yang *et al.*, 2024 [26]	Responses from ChatGPT and Bard, 20 treatments	GPT, BARD	ChatGPT had an 80% concordance rate with AAOS CPGs, while Bard had 60%
England *et al.*, 2024 [38]	Clinical notes from EHRs, 7485 patients	NLP	95.8% of NLP-derived FVC values were within 5% predicted of PFT equipment values
Love *et al.*, 2011 [54]	EMR notes, billing codes, 2318 patients	NLP with random forest	PPV of 90% at sensitivity of 87% for PsA classification using NLP and coded data
Deng *et al.*, 2024 [12]	Structured EHR data, clinical notes, 472 patients	MetaMap, logistic regression	Identified lupus nephritis phenotype with an F1 score of 0.79 at NU and 0.93 at VUMC
van Leeuwen *et al.*, 2024 [50]	EHRs, 287 patients	AI tool, NLP	Sensitivity of 97.0% in training and 98.0% in validation centres for AAV identification
Román Ivorra *et al.*, 2024 [47]	EHRs, 13 958 patients	EHRead, NLP, ML	Achieved precision of 79.4% for ILD detection and 76.4% for RA detection
Zhao *et al.*, 2020 [43]	EHRs, 7853 patients	NLP, ICD codes, logistic regression	Sensitivity of 0.78, specificity of 0.94 and AUROC of 0.93 for identifying axSpA
Kerr *et al.*, 2015 [45]	Clinical narrative data from EMRs, 2280 patients	NLP system	Compliance rates for gout QIs: QI 1, 92.1%; QI 2, 44.8%; QI 3, 7.7%
Redd *et al.*, 2014 [44]	Structured and unstructured EHR data, 4272 patients	NLP, SVM	Precision of 0.814 and recall of 0.973 for identifying SSc patients at risk for SRC
Oliveira *et al.*, 2024 [35]	Chief complaint notes from emergency department, 8037 CCs	RoBERTa-large, BioGPT	Achieved F1 scores of 0.8 (2019 dataset) and 0.85 (2020 dataset) for detecting gout flares
Gräf *et al.*, 2022 [28]	Survey data, clinical vignettes, 132 vignettes	ADA	ADA’s diagnostic accuracy for IRD was higher compared with physicians (70% *vs* 54%)

CCs: Clinical Cases; NER: named entity recognition; POS: parts of speech; CNN: convolutional neural network; YOLO: You Only Look Once; IRD: inflammatory rheumatic disease; FVC: forced vital capacity; QI: quality indicator; PFT: pulmonary function test; ADE: adverse drug event; RISE: Rheumatology Informatics System for Effectiveness; SRC: scleroderma renal crisis; GPA: granulomatosis with polyangiitis; MPA: microscopic polyangiitis; EGPA: eosinophilic granulomatosis with polyangiitis; ML: machine learning; HCPCS: Healthcare Common Procedure Coding System; LASSO: least absolute shrinkage and selection operator; MAP: maximum a posteriori; RoBERTa: A Robustly Optimized BERT Pretraining Approach; BioGPT: Biomedical Generative Pre-trained Transformer; NU: Northwestern University; VUMC: Vanderbilt University Medical Center; HITEx: Health Information Text Extraction; EHRead: Electronic Health Read; ADA: AI-based symptom checker; FFNN: Feedforward neural network.

**Figure 2.**
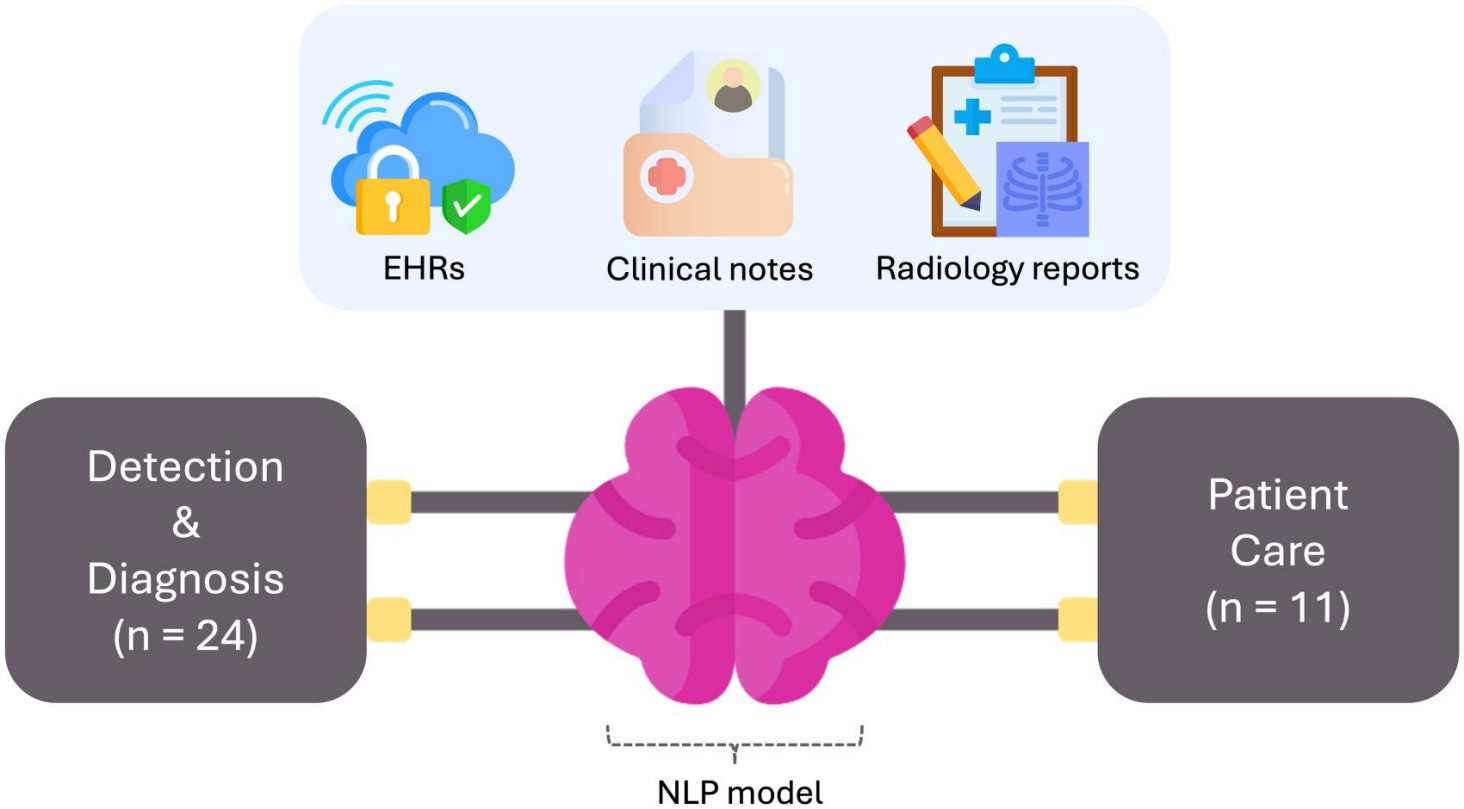
A framework of the NLP model inputs and output categories

The studies covered multiple conditions, including RA (12 studies), SpA (5 studies), gout (5 studies) and other conditions such as lupus, SSc and ANCA-associated vasculitis (AAV; 13 studies) (Fig. 2). Most of the included articles were published in quartile 1 journals (Supplementary Fig. S1, available at *Rheumatology Advances in Practice* online).

### Risk of bias

The analysis of the risk of bias yielded mostly good and fair results using the Quality Assessment Tool for Observational Cohort and Cross-Sectional Studies. Specifically, 20 studies were evaluated as having good quality and low risk of bias, 9 studies as having fair quality and fair risk of bias and 6 studies as having poor quality and high risk of bias. The poor evaluations were mainly due to the use of vignettes or question-based studies that did not fit well under the tool’s evaluation categories. Nonetheless, the overall results indicate a general trend of high quality and low overall risk of bias. A detailed evaluation for each study is provided in Supplementary Table S1 (available at *Rheumatology Advances in Practice* online).

### A background on NLP for clinicians

**Figure 3.**
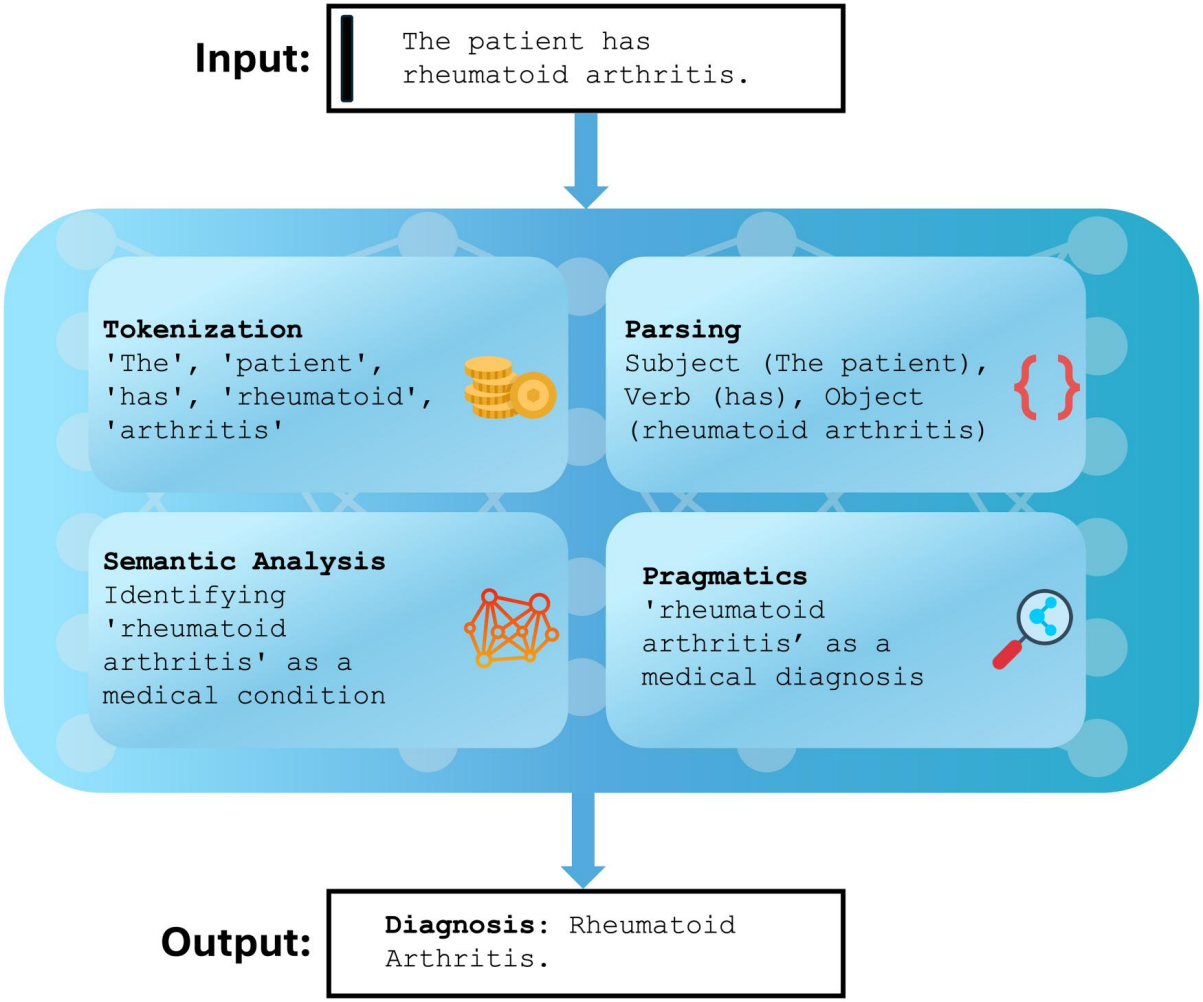
A simple NLP framework in rheumatology

NLP allows machines to interpret and manipulate human language [55]. Key processes include tokenization (breaking text into words and phrases), parsing (analysing sentence structure), semantic analysis (interpreting meaning) and pragmatics (understanding context) [55]. NLP uses statistical analysis, machine learning and deep learning to perform tasks like translation, sentiment analysis and summarization [55, 56] (Fig. 3).

Transformers have revolutionized NLP by enabling parallel processing of input data, improving efficiency and speed [57]. They use self-attention mechanisms to weigh each part of the input independently, enhancing their ability to understand and generate contextually relevant responses [58]. This architecture underlies major models like OpenAI’s GPT series and Google’s BERT [58].

LLMs are trained on extensive text corpora to analyse and generate human-like text [2, 15]. They excel in applications such as automated dialogue systems, content creation and complex analytical tasks [2, 15]. OpenAI’s GPT series is known for generating coherent and context-aware text sequences, thanks to extensive pre-training and fine-tuning [31]. Another example is Meta’s LLaMA, which is an efficient, open-source model available in multiple configurations, while Google’s Gemini, formerly Bard, is designed for high-quality interactions using up-to-date content [59].

In AI, a ‘prompt’ is the input given to a language model to guide its output [2, 8]. Autoregression involves predicting the next word or sequence based on previous inputs, ensuring coherent and contextually appropriate text. This technique is crucial for tasks like text completion and machine translation [2, 8].

### NLP in detecting and diagnosing rheumatic diseases

**Figure 4.**
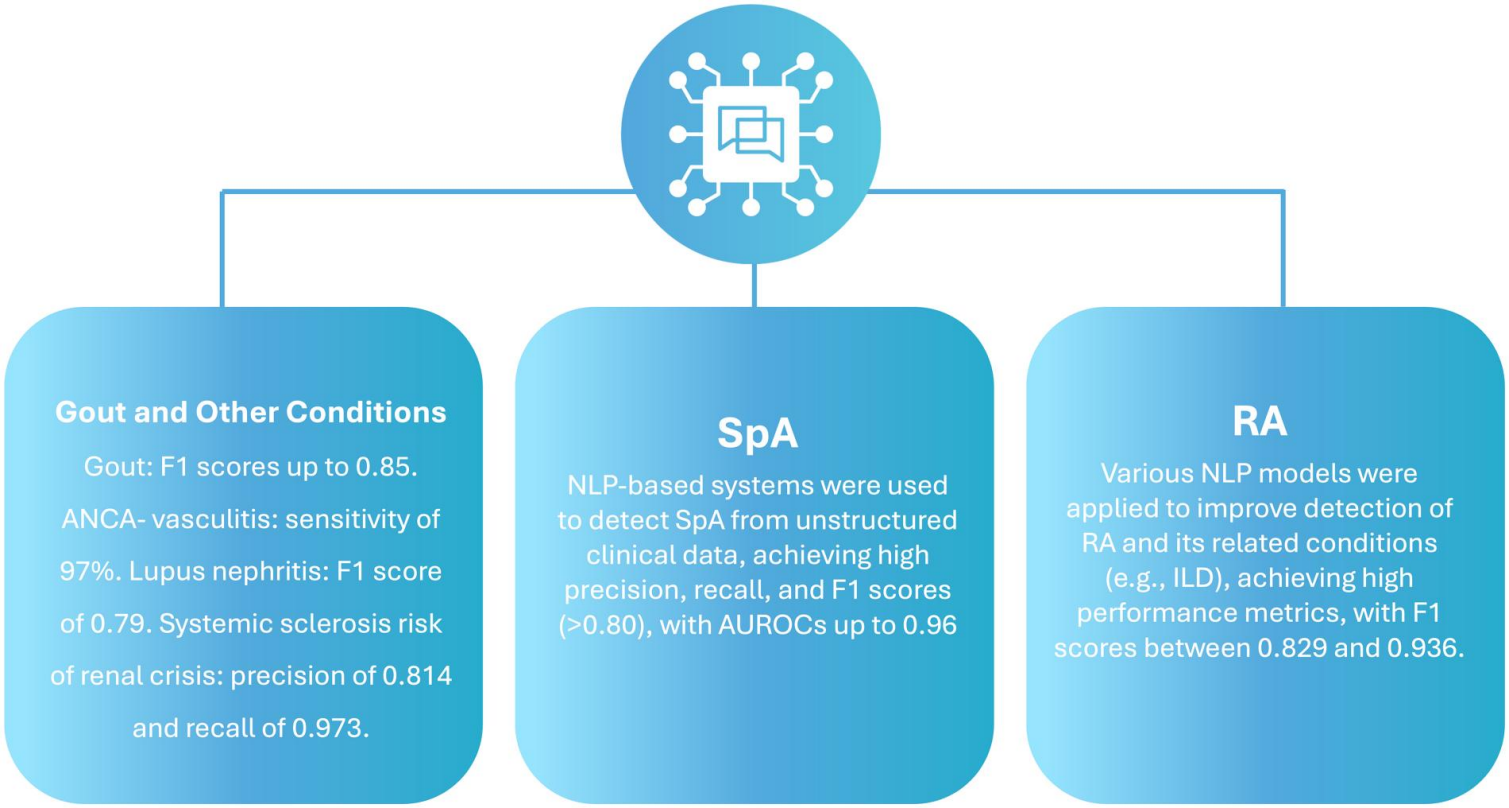
A summary of the applications and performance of NLP models in the detection across different conditions

Overall, 24 studies fell under this application. Specifically, nine studies focused on RA, four on SpA and the others on diseases such as gout, SSc, OA and AAV (Table 1, Fig. 4).

### RA

Various NLP models were used to improve detection and diagnosis of RA. These models were applied to tasks such as named entity recognition, adverse drug event detection and disease activity extraction, achieving high performance metrics with F1 scores up to 0.936, positive predictive values (PPVs) of up to 94% and sensitivities of up to 95%. For instance, Li *et al*. [46] used a BERT-based model to improve named entity recognition in clinical notes, achieving an F1 score of 0.936. Nelson *et al.* [49] demonstrated that NLP significantly improved sensitivity (86%) and PPV (98%) for identifying infliximab infusion dates and doses compared with using international Classification of Diseases (ICD) codes.

Liu *et al.* [25] used BERT for named entity and extraction from Chinese EMRs, achieving F1 scores of 93% for entity recognition and 95% for relation extraction.

Humbert-Droz *et al.* [30] developed an NLP pipeline that showed good internal and external validity for extracting RA disease activity and functional status scores, with sensitivity, PPV and F1 scores of 95%, 87% and 91%, respectively, in internal validation and 92%, 69% and 79%, respectively, in external validation.

Liao *et al.* [36] used the Health Information Text Extraction (HITEx) system, which improved the classification accuracy of RA subjects, compared with using ICD codes, achieving a PPV of 94% using both narrative and codified data. Lin *et al.* [24] combined Apache cTAKES for feature extraction with supervised machine learning, achieving a PPV of 76%, sensitivity of 92% and F1 score of 83% for identifying methotrexate-induced liver toxicity.

Wang *et al.* [32] used NLP tools to discover and validate adverse drug events, detecting 152 signals for biologics and 147 for DMARDs from clinical notes, that were not detected using other traditional tools. Luedders *et al.* [37] used automated regular expressions to enhance RA interstitial lung disease (RA-ILD) identification, achieving a PPV of 95% in the derivation cohort and 89% in the validation cohort.

Similarly, Román Ivorra *et al.* [47] used the EHRead technology to extract and standardize unstructured clinical information to estimate the prevalence of RA-ILD, achieving precisions of 79% for ILD detection and 76% for RA detection. England *et al.* [38] extracted forced vital capacity values from EHR notes, showing that 96% of NLP-derived values were within 5% of predicted pulmonary function test equipment values.

### SpAs

Most of the studies focused on detecting SpAs from unstructured clinical data. For instance, Benavent *et al.* [5] used an NLP-based system to extract and identify clinical entities related to SpA, achieving high precision, recall and F1 scores (>0.80). Walsh *et al*. [40] developed three algorithms for identifying axial SpA (axSpA) from EHRs, with the full algorithm achieving an area under the receiver operating characteristics (AUROC) curve of 0.96, sensitivities of 85–95% and specificities of 78–94%. Zhao *et al.* [43] combined NLP with ICD codes and logistic regression models, achieving an AUROC of 0.93, sensitivity of 78% and specificity of 94% for identifying axSpA. In addition, Love *et al.* [54] focused on using NLP to classify PsA cases from EMRs. Their study showed that using NLP with EMR text notes significantly improved the performance of the prediction algorithm for PsA classification compared with using only coded data. Specifically, the AUROC) improved from 0.925 (coded data alone) to 0.950 (combined coded and NLP data), indicating a significant enhancement in classification accuracy.

### Gout

All the studies focused on detecting gout flares using different data inputs and models. Zheng *et al.* [51] used NLP and machine learning to identify gout flares from unstructured EHR data, achieving a sensitivity of 82% and specificity of 92%. Yoshida *et al*. [42] combined NLP concepts with Medicare claims data, resulting in an AUROC of 0.731 for identifying gout flares. Osborne *et al.* [41] used a BERT-based algorithm to identify gout flares in emergency department patients, achieving an F1 score of 0.57. Oliveira *et al.* [35] compared different models for early detection of gout flares from chief complaint notes, with RoBERTa-large-PM-M3-Voc achieving an F1 score of 0.8 and BioGPT achieving an F1 score of 0.85.

### Other conditions

Other studies addressed various rheumatologic conditions. Deng *et al.* [12] used MetaMap-based models to identify lupus nephritis phenotypes, achieving an F-measure of 0.79 at Northwestern Medicine and 0.93 at Vanderbilt University. Van Leeuwen *et al.* [50] used an AI tool incorporating NLP to identify AAV, achieving sensitivities of 97% and 98% in training and validation centres, respectively. Redd *et al.* [44] used NLP combined with a support vector machine (SVM) to detect SSc patients at risk for scleroderma renal crisis, achieving a precision of 0.814 and recall of 0.973.

### Patient care

**Figure 5.**
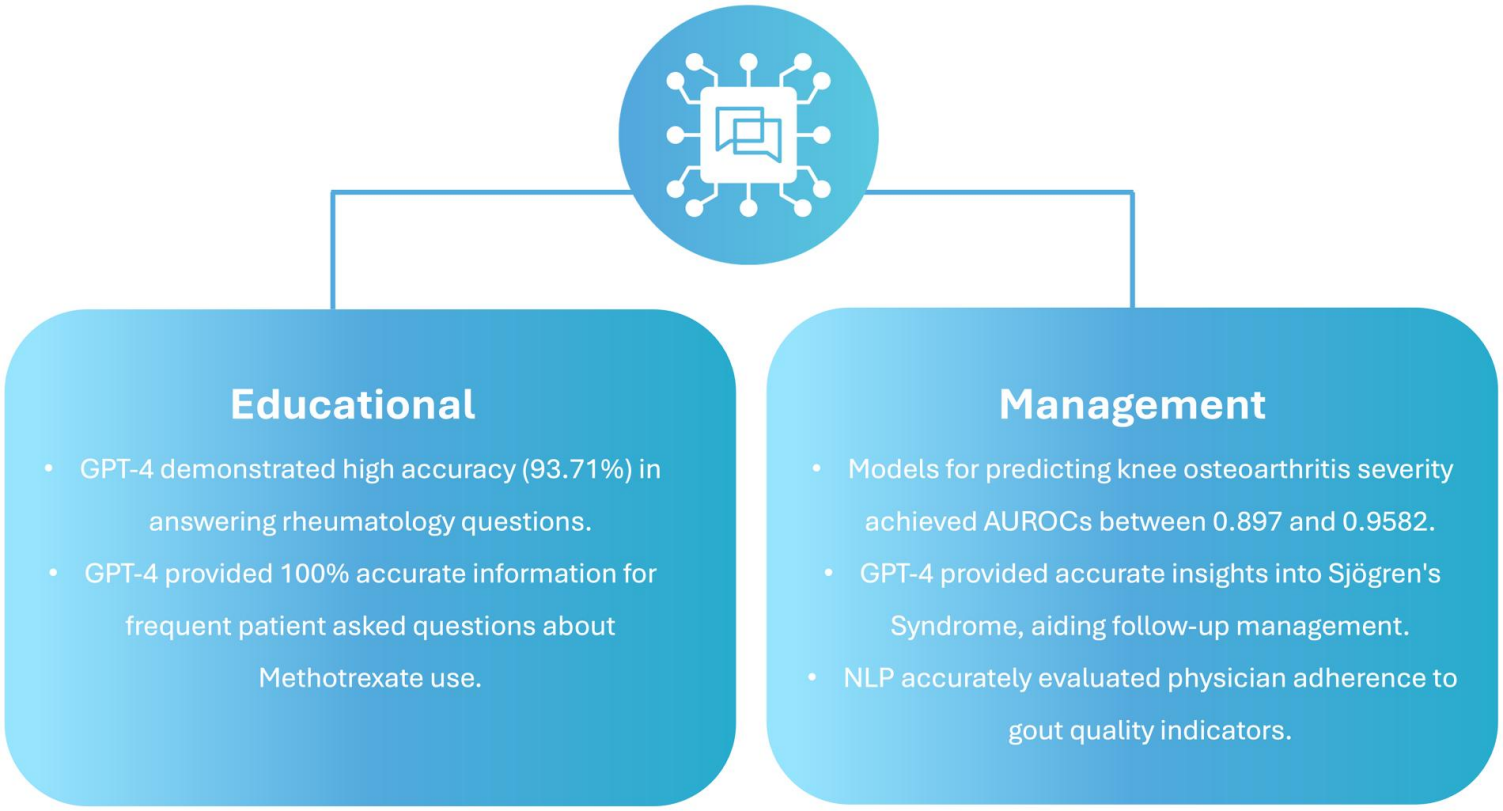
NLP model capabilities in performing high-level research questions under patient care applications

Patient care includes studies focusing on management, educational purposes for patients or practitioners and research. Under this category, there were 11 studies, divided into two main categories: management (plans, treatment, risk stratification, prediction) and education (answering questions, aiding research) (Table 1, Fig. 5).

### Management

Saini *et al.* [29] developed an ensemble model for knee OA severity prediction and report generation, achieving AUROCs from 0.897 to 0.958. Irfan *et al.* [27] used GPT-4 to provide insights into SS, highlighting key immunopathological and histopathological characteristics and providing follow-up management and differential diagnosis. Benavent *et al*. [6] used EHRead technology to explore the characteristics and management of patients with axSpA and PsA, achieving a precision of 0.798 and recall of 0.735 for PsA.

Ye *et al.* [33] compared AI-generated responses to rheumatology patient questions with physician responses. Patients rated AI responses similarly to physician responses, while rheumatologists rated AI responses lower in comprehensiveness and accuracy. Kerr *et al.* [45] used NLP to evaluate physician adherence to gout quality indicators (QIs), finding compliance rates of 92% for QI 1, 45% for QI 2 and 8% for QI 3. Rheumatology clinic visits were associated with greater compliance.

### Educational

Madrid-García *et al.* [39] assessed the accuracy of GPT-4 in answering rheumatology questions, finding an accuracy of 94% and a median clinical reasoning score of 4.7. VanSchaik *et al.* [53] used an ELECTRA-based model to extract causal relationships from biomedical literature, achieving an F1 score of 0.91. Li *et al*. [52] used advanced models like BERT, RoBERTa, ALBERT and MacBERT for question matching in an RA question-answering system, achieving a top-1 precision of 55% and a mean reciprocal rank (MRR) of 0.660.

Coskun *et al.* [23] evaluated the accuracy of AI models in providing information on methotrexate use. GPT-4 achieved 100% accuracy, while GPT-3.5 scored 87%.

Uz and Umay [34] assessed the reliability and usefulness of ChatGPT for common rheumatic disease–related queries. Reliability scores ranged from 4 to 7, with the highest score for OA (5.6), and usefulness scores ranged from 4 to 7, with the highest for AS (5.9). Yang *et al.* [26] evaluated the concordance of ChatGPT and Bard with AAOS CPGs, finding that ChatGPT had an 80% concordance rate and Bard’s was 60%.

## Discussion

NLP technology is starting to influence the management and diagnosis of rheumatic diseases. BERT and GPT, for instance, are showing promise in enhancing diagnostic accuracy for conditions such as RA and SpA [30, 54]. These models deliver results that suggest potential improvements over traditional diagnostic methods, offering better precision.

In clinical environments, NLP has begun to improve interactions between patients and healthcare providers and to augment educational resources for medical professionals [60, 61]. This technology challenges the idea that digital tools necessarily depersonalize care, indicating instead that they can foster more informed and engaging healthcare interactions. This is reflected in our review by studies such as that of Coskun *et al.* [23], which demonstrated the utility of GPT-4 in answering patient questions related to methotrexate use. Additionally, Venerito *et al.* [62] compared multiple LLMs, finding GPT-4 and Claude 2 performed well in answering clinical trivia, indicating their potential in clinical education and decision support, similar to the findings in our review regarding the performance of NLP models in educational applications. Maarseveen *et al.* [63] demonstrated the effectiveness of machine learning algorithms in accurately identifying patients with RA from unstructured text in EHRs. This approach showcases the potential of augmenting NLP models and classical machine learning in rheumatology to potentially enhance patient identification and facilitate large-scale observational studies across different healthcare systems. Ayer *et al.* [60] evaluated AI chatbot responses to patient questions, finding them to be of higher quality and more empathetic than physician responses. Another interesting area for educational use of LLMs was highlighted in recent findings by Haase *et al.* [64]. Their study showed that GPT-4 outperformed SLE experts in providing high-quality, empathetic responses to patient questions. This demonstrates GPT-4’s potential as a valuable tool for enhancing patient education and communication.

NLP also supports the development of personalized treatment plans and advanced disease management, providing alternatives to the traditional one-size-fits-all treatment approaches [65]. This emerging application invites a re-evaluation of established treatment paradigms. Our results suggest that NLP tools can effectively screen patients for comorbidities and associated diseases, such as detecting RA-ILD and extracting vital capacities of RA patients from large amounts of unstructured data [37, 38]. Additionally, these tools can predict or detect flares, enhancing their ability to provide timely and individualized interventions and treatments [29, 35].

Despite the promising results, there is a lack of research on certain rheumatic conditions, especially rare diseases such as Behçet’s disease. Conditions like SSc and lupus nephritis, although somewhat researched, are studied to a lesser extent than diseases like RA and SpA. However, current results suggest that integrating NLP can treat flairs by accurately predicting them, indicating an area for future exploration (Supplementary Table S2, available at *Rheumatology Advances in Practice* online). Expanding the scope of NLP research to cover less common rheumatic conditions and diverse patient demographics could increase the relevance and applicability of NLP tools, potentially challenging the prevailing focus on more prevalent conditions.

Several unmet clinical needs in rheumatology remain unaddressed by current NLP and LLM applications. For instance, preventing complications like falls in RA and cardiovascular disease in SLE is challenging [66, 67]. This technology could potentially contribute to risk stratification and personalized preventive interventions by analysing complex patient data and identifying high-risk individuals. Moreover, these models could aid in distinguishing between overlapping conditions like fibromyalgia and inflammatory arthritis, where patients often present with similar symptoms [68]. By integrating text analysis with clinical and laboratory data, LLMs might discern subtle patterns that could guide diagnosis and treatment decisions [68]. Furthermore, rare diseases like Behçet’s disease pose diagnostic challenges due to their heterogeneous presentations [69]. Advanced models integrating diverse data sources, including family history, demographics, clinical features and genetic markers like HLA-B51, could potentially improve diagnostic accuracy and facilitate early intervention [69].

For NLP to become integral to routine clinical practice, extensive clinical validation is necessary [61]. The current enthusiasm for the capabilities of NLP must be tempered with rigorous, evidence-based trials to bridge the gap between theoretical potential and practical utility. Moreover, the computational intensity required to run advanced NLP models is a significant barrier [70]. This challenge necessitates a balanced approach to technology adoption that considers existing infrastructural limits [71]. Nonetheless, the internet interface is widely available and easily usable, in addition to the use of application programming interfaces for streamlining different applications more efficiently [70, 71]. This could imply a future where these models can be relatively easily implemented and used.

Deploying NLP technologies also raises important ethical and privacy issues [72]. It is crucial to manage data responsibly and enforce stringent privacy measures to maintain trust and integrity within healthcare practices.

In conclusion, NLP shows significant potential to enhance rheumatology by improving diagnostic accuracy and personalizing patient care, particularly in detecting diseases and conditions from unstructured reports, especially for RA, SpA and gout. However, the realization of this potential is still in its early stages. Achieving the full benefits of NLP will require overcoming existing limitations through focused research, ethical commitment and ongoing technological enhancements.

## Supplementary Material

rkae120_Supplementary_Data

## Data Availability

The data underlying this article will be available upon reasonable request to the corresponding author.
