# Supplementary material for: Advancing rheumatology with natural language processing: insights and prospects from a systematic review
Source: Rheumatol Adv Pract. 2024 Sep 19;8(4):rkae120. doi: 10.1093/rap/rkae120 (PMC11467191; doi:10.1093/rap/rkae120)
Supplement: rkae120_Supplementary_Data [file rkae120_supplementary_data.zip › RHEUMAP-2024-125.R1 - Supplementary material.docx]

**Supplementary materials**

**Adapting Rheumatology to the Accelerating Pace of NLP: Systematic Review of Current Applications and Future Directions**

Boolean strings used for each database

**PubMed**

| **Query** | **Filters** |  | **Results** |
| --- | --- | --- | --- |
| ((("Natural Language Processing"[Title/Abstract] OR "NLP"[Title/Abstract] OR "Language Model"[Title/Abstract] OR "Large Language Model"[Title/Abstract] OR "LLM"[Title/Abstract] OR "LLMs"[Title/Abstract] OR "Generative Pre-trained Transformer"[Title/Abstract] OR "GPT"[Title/Abstract] OR "GPT-4"[Title/Abstract] OR "GPT-3.5"[Title/Abstract] OR "GPT-3"[Title/Abstract] OR "ChatGPT"[Title/Abstract] OR "BERT"[Title/Abstract] OR "XLNet"[Title/Abstract] OR "RoBERTa"[Title/Abstract] OR "ELECTRA"[Title/Abstract] OR "ALBERT"[Title/Abstract] OR "Megatron"[Title/Abstract] OR "Grok"[Title/Abstract] OR "Galactica"[Title/Abstract] OR "Gemini"[Title/Abstract] OR "Claude"[Title/Abstract] OR "LLaMA"[Title/Abstract] OR "PaLM"[Title/Abstract])) AND (("Rheumatology"[Title/Abstract] OR "Rheumatic Disease"[Title/Abstract] OR "Arthritis"[Title/Abstract] OR "Rheumatoid Arthritis"[Title/Abstract] OR "Osteoarthritis"[Title/Abstract] OR "Psoriatic Arthritis"[Title/Abstract] OR "Ankylosing Spondylitis"[Title/Abstract] OR "Lupus"[Title/Abstract] OR "Systemic Lupus Erythematosus"[Title/Abstract] OR "Scleroderma"[Title/Abstract] OR "Sjogren's Syndrome"[Title/Abstract] OR "Gout"[Title/Abstract] OR "Vasculitis"[Title/Abstract] OR "Juvenile Idiopathic Arthritis"[Title/Abstract] OR "Fibromyalgia"[Title/Abstract] OR "Polymyalgia Rheumatica"[Title/Abstract] OR "Dermatomyositis"[Title/Abstract] OR "Polymyositis"[Title/Abstract] OR "Mixed Connective Tissue Disease"[Title/Abstract] OR "Behcet's Disease"[Title/Abstract]))) | English, from 2002 - 2024 | (("Natural Language Processing"[Title/Abstract] OR "NLP"[Title/Abstract] OR "Language Model"[Title/Abstract] OR "Large Language Model"[Title/Abstract] OR "LLM"[Title/Abstract] OR "LLMs"[Title/Abstract] OR "Generative Pre-trained Transformer"[Title/Abstract] OR "GPT"[Title/Abstract] OR "GPT-4"[Title/Abstract] OR "GPT-3.5"[Title/Abstract] OR "GPT-3"[Title/Abstract] OR "ChatGPT"[Title/Abstract] OR "BERT"[Title/Abstract] OR "XLNet"[Title/Abstract] OR "RoBERTa"[Title/Abstract] OR "ELECTRA"[Title/Abstract] OR "ALBERT"[Title/Abstract] OR "Megatron"[Title/Abstract] OR "Grok"[Title/Abstract] OR "Galactica"[Title/Abstract] OR "Gemini"[Title/Abstract] OR "Claude"[Title/Abstract] OR "LLaMA"[Title/Abstract] OR "PaLM"[Title/Abstract]) AND ("Rheumatology"[Title/Abstract] OR "Rheumatic Disease"[Title/Abstract] OR "Arthritis"[Title/Abstract] OR "Rheumatoid Arthritis"[Title/Abstract] OR "Osteoarthritis"[Title/Abstract] OR "Psoriatic Arthritis"[Title/Abstract] OR "Ankylosing Spondylitis"[Title/Abstract] OR "Lupus"[Title/Abstract] OR "Systemic Lupus Erythematosus"[Title/Abstract] OR "Scleroderma"[Title/Abstract] OR "Sjogren's Syndrome"[Title/Abstract] OR "Gout"[Title/Abstract] OR "Vasculitis"[Title/Abstract] OR "Juvenile Idiopathic Arthritis"[Title/Abstract] OR "Fibromyalgia"[Title/Abstract] OR "Polymyalgia Rheumatica"[Title/Abstract] OR "Dermatomyositis"[Title/Abstract] OR "Polymyositis"[Title/Abstract] OR "Mixed Connective Tissue Disease"[Title/Abstract] OR "Behcet's Disease"[Title/Abstract])) AND ((english[Filter]) AND (2002:2024[pdat])) | 338 |
| ((("Natural Language Processing"[Title/Abstract] OR "NLP"[Title/Abstract] OR "Language Model"[Title/Abstract] OR "Large Language Model"[Title/Abstract] OR "LLM"[Title/Abstract] OR "LLMs"[Title/Abstract] OR "Generative Pre-trained Transformer"[Title/Abstract] OR "GPT"[Title/Abstract] OR "GPT-4"[Title/Abstract] OR "GPT-3.5"[Title/Abstract] OR "GPT-3"[Title/Abstract] OR "ChatGPT"[Title/Abstract] OR "BERT"[Title/Abstract] OR "XLNet"[Title/Abstract] OR "RoBERTa"[Title/Abstract] OR "ELECTRA"[Title/Abstract] OR "ALBERT"[Title/Abstract] OR "Megatron"[Title/Abstract] OR "Grok"[Title/Abstract] OR "Galactica"[Title/Abstract] OR "Gemini"[Title/Abstract] OR "Claude"[Title/Abstract] OR "LLaMA"[Title/Abstract] OR "PaLM"[Title/Abstract])) AND (("Rheumatology"[Title/Abstract] OR "Rheumatic Disease"[Title/Abstract] OR "Arthritis"[Title/Abstract] OR "Rheumatoid Arthritis"[Title/Abstract] OR "Osteoarthritis"[Title/Abstract] OR "Psoriatic Arthritis"[Title/Abstract] OR "Ankylosing Spondylitis"[Title/Abstract] OR "Lupus"[Title/Abstract] OR "Systemic Lupus Erythematosus"[Title/Abstract] OR "Scleroderma"[Title/Abstract] OR "Sjogren's Syndrome"[Title/Abstract] OR "Gout"[Title/Abstract] OR "Vasculitis"[Title/Abstract] OR "Juvenile Idiopathic Arthritis"[Title/Abstract] OR "Fibromyalgia"[Title/Abstract] OR "Polymyalgia Rheumatica"[Title/Abstract] OR "Dermatomyositis"[Title/Abstract] OR "Polymyositis"[Title/Abstract] OR "Mixed Connective Tissue Disease"[Title/Abstract] OR "Behcet's Disease"[Title/Abstract]))) | from 2002 - 2024 | (("Natural Language Processing"[Title/Abstract] OR "NLP"[Title/Abstract] OR "Language Model"[Title/Abstract] OR "Large Language Model"[Title/Abstract] OR "LLM"[Title/Abstract] OR "LLMs"[Title/Abstract] OR "Generative Pre-trained Transformer"[Title/Abstract] OR "GPT"[Title/Abstract] OR "GPT-4"[Title/Abstract] OR "GPT-3.5"[Title/Abstract] OR "GPT-3"[Title/Abstract] OR "ChatGPT"[Title/Abstract] OR "BERT"[Title/Abstract] OR "XLNet"[Title/Abstract] OR "RoBERTa"[Title/Abstract] OR "ELECTRA"[Title/Abstract] OR "ALBERT"[Title/Abstract] OR "Megatron"[Title/Abstract] OR "Grok"[Title/Abstract] OR "Galactica"[Title/Abstract] OR "Gemini"[Title/Abstract] OR "Claude"[Title/Abstract] OR "LLaMA"[Title/Abstract] OR "PaLM"[Title/Abstract]) AND ("Rheumatology"[Title/Abstract] OR "Rheumatic Disease"[Title/Abstract] OR "Arthritis"[Title/Abstract] OR "Rheumatoid Arthritis"[Title/Abstract] OR "Osteoarthritis"[Title/Abstract] OR "Psoriatic Arthritis"[Title/Abstract] OR "Ankylosing Spondylitis"[Title/Abstract] OR "Lupus"[Title/Abstract] OR "Systemic Lupus Erythematosus"[Title/Abstract] OR "Scleroderma"[Title/Abstract] OR "Sjogren's Syndrome"[Title/Abstract] OR "Gout"[Title/Abstract] OR "Vasculitis"[Title/Abstract] OR "Juvenile Idiopathic Arthritis"[Title/Abstract] OR "Fibromyalgia"[Title/Abstract] OR "Polymyalgia Rheumatica"[Title/Abstract] OR "Dermatomyositis"[Title/Abstract] OR "Polymyositis"[Title/Abstract] OR "Mixed Connective Tissue Disease"[Title/Abstract] OR "Behcet's Disease"[Title/Abstract])) AND (2002:2024[pdat]) | 359 |
| ((("Natural Language Processing"[Title/Abstract] OR "NLP"[Title/Abstract] OR "Language Model"[Title/Abstract] OR "Large Language Model"[Title/Abstract] OR "LLM"[Title/Abstract] OR "LLMs"[Title/Abstract] OR "Generative Pre-trained Transformer"[Title/Abstract] OR "GPT"[Title/Abstract] OR "GPT-4"[Title/Abstract] OR "GPT-3.5"[Title/Abstract] OR "GPT-3"[Title/Abstract] OR "ChatGPT"[Title/Abstract] OR "BERT"[Title/Abstract] OR "XLNet"[Title/Abstract] OR "RoBERTa"[Title/Abstract] OR "ELECTRA"[Title/Abstract] OR "ALBERT"[Title/Abstract] OR "Megatron"[Title/Abstract] OR "Grok"[Title/Abstract] OR "Galactica"[Title/Abstract] OR "Gemini"[Title/Abstract] OR "Claude"[Title/Abstract] OR "LLaMA"[Title/Abstract] OR "PaLM"[Title/Abstract])) AND (("Rheumatology"[Title/Abstract] OR "Rheumatic Disease"[Title/Abstract] OR "Arthritis"[Title/Abstract] OR "Rheumatoid Arthritis"[Title/Abstract] OR "Osteoarthritis"[Title/Abstract] OR "Psoriatic Arthritis"[Title/Abstract] OR "Ankylosing Spondylitis"[Title/Abstract] OR "Lupus"[Title/Abstract] OR "Systemic Lupus Erythematosus"[Title/Abstract] OR "Scleroderma"[Title/Abstract] OR "Sjogren's Syndrome"[Title/Abstract] OR "Gout"[Title/Abstract] OR "Vasculitis"[Title/Abstract] OR "Juvenile Idiopathic Arthritis"[Title/Abstract] OR "Fibromyalgia"[Title/Abstract] OR "Polymyalgia Rheumatica"[Title/Abstract] OR "Dermatomyositis"[Title/Abstract] OR "Polymyositis"[Title/Abstract] OR "Mixed Connective Tissue Disease"[Title/Abstract] OR "Behcet's Disease"[Title/Abstract]))) |  | ("Natural Language Processing"[Title/Abstract] OR "NLP"[Title/Abstract] OR "Language Model"[Title/Abstract] OR "Large Language Model"[Title/Abstract] OR "LLM"[Title/Abstract] OR "LLMs"[Title/Abstract] OR "Generative Pre-trained Transformer"[Title/Abstract] OR "GPT"[Title/Abstract] OR "GPT-4"[Title/Abstract] OR "GPT-3.5"[Title/Abstract] OR "GPT-3"[Title/Abstract] OR "ChatGPT"[Title/Abstract] OR "BERT"[Title/Abstract] OR "XLNet"[Title/Abstract] OR "RoBERTa"[Title/Abstract] OR "ELECTRA"[Title/Abstract] OR "ALBERT"[Title/Abstract] OR "Megatron"[Title/Abstract] OR "Grok"[Title/Abstract] OR "Galactica"[Title/Abstract] OR "Gemini"[Title/Abstract] OR "Claude"[Title/Abstract] OR "LLaMA"[Title/Abstract] OR "PaLM"[Title/Abstract]) AND ("Rheumatology"[Title/Abstract] OR "Rheumatic Disease"[Title/Abstract] OR "Arthritis"[Title/Abstract] OR "Rheumatoid Arthritis"[Title/Abstract] OR "Osteoarthritis"[Title/Abstract] OR "Psoriatic Arthritis"[Title/Abstract] OR "Ankylosing Spondylitis"[Title/Abstract] OR "Lupus"[Title/Abstract] OR "Systemic Lupus Erythematosus"[Title/Abstract] OR "Scleroderma"[Title/Abstract] OR "Sjogren's Syndrome"[Title/Abstract] OR "Gout"[Title/Abstract] OR "Vasculitis"[Title/Abstract] OR "Juvenile Idiopathic Arthritis"[Title/Abstract] OR "Fibromyalgia"[Title/Abstract] OR "Polymyalgia Rheumatica"[Title/Abstract] OR "Dermatomyositis"[Title/Abstract] OR "Polymyositis"[Title/Abstract] OR "Mixed Connective Tissue Disease"[Title/Abstract] OR "Behcet's Disease"[Title/Abstract]) | 453 |
| ("Natural Language Processing" OR "NLP" OR "Language Model" OR "Large Language Model" OR "LLM" OR "LLMs" OR "Generative Pre-trained Transformer" OR "GPT" OR "GPT-4" OR "GPT-3.5" OR "GPT-3" OR ChatGPT OR "BERT" OR "XLNet" OR "RoBERTa" OR "ELECTRA" OR "ALBERT" OR "Megatron" OR "Grok" OR "Galactica" OR "Gemini" OR "Claude" OR "LLaMA" OR "PaLM") AND ("Rheumatology" OR "Rheumatic Disease" OR "Arthritis" OR "Rheumatoid Arthritis" OR "Osteoarthritis" OR "Psoriatic Arthritis" OR "Ankylosing Spondylitis" OR "Lupus" OR "Systemic Lupus Erythematosus" OR "Scleroderma" OR "Sjogren's Syndrome" OR "Gout" OR "Vasculitis" OR "Juvenile Idiopathic Arthritis" OR "Fibromyalgia" OR "Polymyalgia Rheumatica" OR "Dermatomyositis" OR "Polymyositis" OR "Mixed Connective Tissue Disease" OR "Behcet's Disease") |  | ("Natural Language Processing"[All Fields] OR "NLP"[All Fields] OR "Language Model"[All Fields] OR "Large Language Model"[All Fields] OR "LLM"[All Fields] OR "LLMs"[All Fields] OR "Generative Pre-trained Transformer"[All Fields] OR "GPT"[All Fields] OR "GPT-4"[All Fields] OR "GPT-3.5"[All Fields] OR "GPT-3"[All Fields] OR "ChatGPT"[All Fields] OR "BERT"[All Fields] OR "XLNet"[All Fields] OR "RoBERTa"[All Fields] OR "ELECTRA"[All Fields] OR "ALBERT"[All Fields] OR "Megatron"[All Fields] OR "Grok"[All Fields] OR "Galactica"[All Fields] OR "Gemini"[All Fields] OR "Claude"[All Fields] OR "LLaMA"[All Fields] OR "PaLM"[All Fields]) AND ("Rheumatology"[All Fields] OR "Rheumatic Disease"[All Fields] OR "Arthritis"[All Fields] OR "Rheumatoid Arthritis"[All Fields] OR "Osteoarthritis"[All Fields] OR "Psoriatic Arthritis"[All Fields] OR "Ankylosing Spondylitis"[All Fields] OR "Lupus"[All Fields] OR "Systemic Lupus Erythematosus"[All Fields] OR "Scleroderma"[All Fields] OR "Sjogren's Syndrome"[All Fields] OR "Gout"[All Fields] OR "Vasculitis"[All Fields] OR "Juvenile Idiopathic Arthritis"[All Fields] OR "Fibromyalgia"[All Fields] OR "Polymyalgia Rheumatica"[All Fields] OR "Dermatomyositis"[All Fields] OR "Polymyositis"[All Fields] OR "Mixed Connective Tissue Disease"[All Fields] OR "Behcet's Disease"[All Fields]) | 5,151 |
| ("Natural Language Processing" OR "NLP" OR "Language Model" OR "Large Language Model" OR "LLM" OR "LLMs" OR "Generative Pre-trained Transformer" OR "GPT" OR "GPT-4" OR "GPT-3.5" OR "GPT-3" OR ChatGPT OR "BERT" OR "XLNet" OR "RoBERTa" OR "ELECTRA" OR "ALBERT" OR "Megatron" OR "Grok" OR "Galactica" OR "Gemini" OR "Claude" OR "LLaMA" OR "PaLM") AND ("Rheumatology" OR "Rheumatic Disease*" OR "Arthritis" OR "Rheumatoid Arthritis" OR "Osteoarthritis" OR "Psoriatic Arthritis" OR "Ankylosing Spondylitis" OR "Lupus" OR "Systemic Lupus Erythematosus" OR "Scleroderma" OR "Sjogren's Syndrome" OR "Gout" OR "Vasculitis" OR "Juvenile Idiopathic Arthritis" OR "Fibromyalgia" OR "Polymyalgia Rheumatica" OR "Dermatomyositis" OR "Polymyositis" OR "Mixed Connective Tissue Disease" OR "Behcet's Disease") |  | ("Natural Language Processing"[All Fields] OR "NLP"[All Fields] OR "Language Model"[All Fields] OR "Large Language Model"[All Fields] OR "LLM"[All Fields] OR "LLMs"[All Fields] OR "Generative Pre-trained Transformer"[All Fields] OR "GPT"[All Fields] OR "GPT-4"[All Fields] OR "GPT-3.5"[All Fields] OR "GPT-3"[All Fields] OR "ChatGPT"[All Fields] OR "BERT"[All Fields] OR "XLNet"[All Fields] OR "RoBERTa"[All Fields] OR "ELECTRA"[All Fields] OR "ALBERT"[All Fields] OR "Megatron"[All Fields] OR "Grok"[All Fields] OR "Galactica"[All Fields] OR "Gemini"[All Fields] OR "Claude"[All Fields] OR "LLaMA"[All Fields] OR "PaLM"[All Fields]) AND ("Rheumatology"[All Fields] OR "rheumatic disease*"[All Fields] OR "Arthritis"[All Fields] OR "Rheumatoid Arthritis"[All Fields] OR "Osteoarthritis"[All Fields] OR "Psoriatic Arthritis"[All Fields] OR "Ankylosing Spondylitis"[All Fields] OR "Lupus"[All Fields] OR "Systemic Lupus Erythematosus"[All Fields] OR "Scleroderma"[All Fields] OR "Sjogren's Syndrome"[All Fields] OR "Gout"[All Fields] OR "Vasculitis"[All Fields] OR "Juvenile Idiopathic Arthritis"[All Fields] OR "Fibromyalgia"[All Fields] OR "Polymyalgia Rheumatica"[All Fields] OR "Dermatomyositis"[All Fields] OR "Polymyositis"[All Fields] OR "Mixed Connective Tissue Disease"[All Fields] OR "Behcet's Disease"[All Fields]) | 5,183 |
| ("Natural Language Processing" OR "NLP" OR "Language Model*" OR "Large Language Model*" OR LLM* OR "Generative Pre-trained Transformer" OR "GPT" OR "GPT-4" OR "GPT-3.5" OR "GPT-3" OR ChatGPT OR "BERT" OR "XLNet" OR "RoBERTa" OR "ELECTRA" OR "ALBERT" OR "Megatron" OR "Grok" OR "Galactica" OR "Gemini" OR "Claude" OR "LLaMA" OR "PaLM") AND ("Rheumatology" OR "Rheumatic Disease*" OR "Arthritis" OR "Rheumatoid Arthritis" OR "Osteoarthritis" OR "Psoriatic Arthritis" OR "Ankylosing Spondylitis" OR "Lupus" OR "Systemic Lupus Erythematosus" OR "Scleroderma" OR "Sjogren's Syndrome" OR "Gout" OR "Vasculitis" OR "Juvenile Idiopathic Arthritis" OR "Fibromyalgia" OR "Polymyalgia Rheumatica" OR "Dermatomyositis" OR "Polymyositis" OR "Mixed Connective Tissue Disease" OR "Behcet's Disease") |  | ("Natural Language Processing"[All Fields] OR "NLP"[All Fields] OR "language model*"[All Fields] OR "large language model*"[All Fields] OR "llm"[All Fields] OR "Generative Pre-trained Transformer"[All Fields] OR "GPT"[All Fields] OR "GPT-4"[All Fields] OR "GPT-3.5"[All Fields] OR "GPT-3"[All Fields] OR "ChatGPT"[All Fields] OR "BERT"[All Fields] OR "XLNet"[All Fields] OR "RoBERTa"[All Fields] OR "ELECTRA"[All Fields] OR "ALBERT"[All Fields] OR "Megatron"[All Fields] OR "Grok"[All Fields] OR "Galactica"[All Fields] OR "Gemini"[All Fields] OR "Claude"[All Fields] OR "LLaMA"[All Fields] OR "PaLM"[All Fields]) AND ("Rheumatology"[All Fields] OR "rheumatic disease*"[All Fields] OR "Arthritis"[All Fields] OR "Rheumatoid Arthritis"[All Fields] OR "Osteoarthritis"[All Fields] OR "Psoriatic Arthritis"[All Fields] OR "Ankylosing Spondylitis"[All Fields] OR "Lupus"[All Fields] OR "Systemic Lupus Erythematosus"[All Fields] OR "Scleroderma"[All Fields] OR "Sjogren's Syndrome"[All Fields] OR "Gout"[All Fields] OR "Vasculitis"[All Fields] OR "Juvenile Idiopathic Arthritis"[All Fields] OR "Fibromyalgia"[All Fields] OR "Polymyalgia Rheumatica"[All Fields] OR "Dermatomyositis"[All Fields] OR "Polymyositis"[All Fields] OR "Mixed Connective Tissue Disease"[All Fields] OR "Behcet's Disease"[All Fields]) | 5,192 |
| ("Natural Language Processing" OR NLP OR "Language Model*" OR "Large Language Model*" OR LLM* OR "Generative Pre-trained Transformer" OR GPT OR "GPT-4" OR "GPT-3.5" OR "GPT-3" OR "GPT-2" OR ChatGPT OR "BERT" OR "T5" OR "XLNet" OR "RoBERTa" OR "ELECTRA" OR "ALBERT" OR "Megatron" OR "Grok" OR "Galactica" OR "Gemini" OR "Claude" OR "BLOOM" OR "LLaMA" OR "ERNIE" OR "PaLM" OR "DeepMind" OR "LaMDA" OR "Anthropic" OR "Mistral" OR "Co:here" OR "Turing-NLG") AND ("Rheumatology" OR "Rheumatic Disease*" OR "Arthritis" OR "Rheumatoid Arthritis" OR "Osteoarthritis" OR "Psoriatic Arthritis" OR "Ankylosing Spondylitis" OR "Lupus" OR "Systemic Lupus Erythematosus" OR "Scleroderma" OR "Sjogren's Syndrome" OR "Gout" OR "Vasculitis" OR "Juvenile Idiopathic Arthritis" OR "Fibromyalgia" OR "Polymyalgia Rheumatica" OR "Dermatomyositis" OR "Polymyositis" OR "Mixed Connective Tissue Disease" OR "Behcet's Disease") |  | ("Natural Language Processing"[All Fields] OR "NLP"[All Fields] OR "language model*"[All Fields] OR "large language model*"[All Fields] OR "llm"[All Fields] OR "Generative Pre-trained Transformer"[All Fields] OR "GPT"[All Fields] OR "GPT-4"[All Fields] OR "GPT-3.5"[All Fields] OR "GPT-3"[All Fields] OR "GPT-2"[All Fields] OR "ChatGPT"[All Fields] OR "BERT"[All Fields] OR "T5"[All Fields] OR "XLNet"[All Fields] OR "RoBERTa"[All Fields] OR "ELECTRA"[All Fields] OR "ALBERT"[All Fields] OR "Megatron"[All Fields] OR "Grok"[All Fields] OR "Galactica"[All Fields] OR "Gemini"[All Fields] OR "Claude"[All Fields] OR "BLOOM"[All Fields] OR "LLaMA"[All Fields] OR "ERNIE"[All Fields] OR "PaLM"[All Fields] OR "DeepMind"[All Fields] OR "LaMDA"[All Fields] OR "Anthropic"[All Fields] OR "Mistral"[All Fields] OR cohere[Author]) AND ("Rheumatology"[All Fields] OR "rheumatic disease*"[All Fields] OR "Arthritis"[All Fields] OR "Rheumatoid Arthritis"[All Fields] OR "Osteoarthritis"[All Fields] OR "Psoriatic Arthritis"[All Fields] OR "Ankylosing Spondylitis"[All Fields] OR "Lupus"[All Fields] OR "Systemic Lupus Erythematosus"[All Fields] OR "Scleroderma"[All Fields] OR "Sjogren's Syndrome"[All Fields] OR "Gout"[All Fields] OR "Vasculitis"[All Fields] OR "Juvenile Idiopathic Arthritis"[All Fields] OR "Fibromyalgia"[All Fields] OR "Polymyalgia Rheumatica"[All Fields] OR "Dermatomyositis"[All Fields] OR "Polymyositis"[All Fields] OR "Mixed Connective Tissue Disease"[All Fields] OR "Behcet's Disease"[All Fields]) | 5,640 |

**OVID-Medline**

(("Natural Language Processing" or "NLP" or "Language Model" or "Large Language Model" or "LLM" or "LLMs" or "Generative Pre-trained Transformer" or "GPT" or "GPT-4" or "GPT-3.5" or "GPT-3" or "ChatGPT" or "BERT" or "XLNet" or "RoBERTa" or "ELECTRA" or "ALBERT" or "Megatron" or "Grok" or "Galactica" or "Gemini" or "Claude" or "LLaMA" or "PaLM") and ("Rheumatology" or "Rheumatic Disease" or "Arthritis" or "Rheumatoid Arthritis" or "Osteoarthritis" or "Psoriatic Arthritis" or "Ankylosing Spondylitis" or "Lupus" or "Systemic Lupus Erythematosus" or "Scleroderma" or "Sjogren's Syndrome" or "Gout" or "Vasculitis" or "Juvenile Idiopathic Arthritis" or "Fibromyalgia" or "Polymyalgia Rheumatica" or "Dermatomyositis" or "Polymyositis" or "Mixed Connective Tissue Disease" or "Behcet's Disease")).ti,ab.

limit 1 to article [Limit not valid in Ovid MEDLINE(R),APA PsycInfo; records were retained]

limit 2 to english language

[BIOSIS Previews <1980 to 2009>](https://ovidsp-dc1-ovid-com.rproxy.tau.ac.il/ovid-new-b/ovidweb.cgi?Titles+Display=G%7CS.sh.47%7C1&S=CGEEFPJKMBACLKEJKPJJJGMIDIAHAA00)

[Ovid MEDLINE(R) and Epub Ahead of Print, In-Process, In-Data-Review & Other Non-Indexed Citations, Daily and Versions <1946 to May 24, 2024>](https://ovidsp-dc1-ovid-com.rproxy.tau.ac.il/ovid-new-b/ovidweb.cgi?Titles+Display=G%7CS.sh.47%7C51&S=CGEEFPJKMBACLKEJKPJJJGMIDIAHAA00)

[APA PsycInfo <1806 to May Week 4 2024>](https://ovidsp-dc1-ovid-com.rproxy.tau.ac.il/ovid-new-b/ovidweb.cgi?Titles+Display=G%7CS.sh.47%7C398&S=CGEEFPJKMBACLKEJKPJJJGMIDIAHAA00)

**Embase**

('natural language processing':ti,ab OR 'nlp':ti,ab OR 'language model':ti,ab OR 'large language model':ti,ab OR 'llm':ti,ab OR 'llms':ti,ab OR 'generative pre-trained transformer':ti,ab OR 'gpt':ti,ab OR 'gpt-4':ti,ab OR 'gpt-3.5':ti,ab OR 'gpt-3':ti,ab OR 'chatgpt':ti,ab OR 'bert':ti,ab OR 'xlnet':ti,ab OR 'roberta':ti,ab OR 'electra':ti,ab OR 'albert':ti,ab OR 'megatron':ti,ab OR 'grok':ti,ab OR 'galactica':ti,ab OR 'gemini':ti,ab OR 'claude':ti,ab OR 'llama':ti,ab OR 'palm':ti,ab) AND ('rheumatology':ti,ab OR 'rheumatic disease':ti,ab OR 'arthritis':ti,ab OR 'rheumatoid arthritis':ti,ab OR 'osteoarthritis':ti,ab OR 'psoriatic arthritis':ti,ab OR 'ankylosing spondylitis':ti,ab OR 'lupus':ti,ab OR 'systemic lupus erythematosus':ti,ab OR 'scleroderma':ti,ab OR 'sjogren syndrome':ti,ab OR 'gout':ti,ab OR 'vasculitis':ti,ab OR 'juvenile idiopathic arthritis':ti,ab OR 'fibromyalgia':ti,ab OR 'polymyalgia rheumatica':ti,ab OR 'dermatomyositis':ti,ab OR 'polymyositis':ti,ab OR 'mixed connective tissue disease':ti,ab OR 'behcet disease':ti,ab) AND (2002:py OR 2003:py OR 2004:py OR 2005:py OR 2006:py OR 2007:py OR 2008:py OR 2009:py OR 2010:py OR 2011:py OR 2012:py OR 2013:py OR 2014:py OR 2015:py OR 2016:py OR 2017:py OR 2018:py OR 2019:py OR 2020:py OR 2021:py OR 2022:py OR 2023:py OR 2024:py) AND [embase]/lim NOT ([embase]/lim AND [medline]/lim) AND 'human'/de AND 'article'/it

**Web of Science**

((TS=("Natural Language Processing" OR "NLP" OR "Language Model" OR "Large Language Model" OR "LLM" OR "LLMs" OR "Generative Pre-trained Transformer" OR "GPT" OR "GPT-4" OR "GPT-3.5" OR "GPT-3" OR "ChatGPT" OR "BERT" OR "XLNet" OR "RoBERTa" OR "ELECTRA" OR "ALBERT" OR "metatron" OR "grow" OR "Galactica" OR "Gemini" OR "Claude" OR "LLaMA" OR "PaLM"))

AND

(TS=("Rheumatology" OR "Rheumatic Disease" OR "Arthritis" OR "Rheumatoid Arthritis" OR "Osteoarthritis" OR "Psoriatic Arthritis" OR "Ankylosing Spondylitis" OR "Lupus" OR "Systemic Lupus Erythematosus" OR "Scleroderma" OR "Sjogren's Syndrome" OR "Gout" OR "Vasculitis" OR "Juvenile Idiopathic Arthritis" OR "Fibromyalgia" OR "Polymyalgia Rheumatica" OR "Dermatomyositis" OR "Polymyositis" OR "Mixed Connective Tissue Disease" OR "Behcet's Disease"))) and 2002 or 2003 or 2004 or 2005 or 2006 or 2007 or 2008 or 2009 or 2010 or 2011 or 2012 or 2013 or 2014 or 2015 or 2016 or 2017 or 2018 or 2019 or 2020 or 2021 or 2022 or 2023 or 2024 (Publication Years) and Article (Document Types) and English (Languages)

**IEEE Xplore**

("natural language processing" OR "NLP" OR "large language models" OR "LLM" OR "BERT" OR "GPT-3" OR "GPT" OR "ChatGPT" OR "GPT-3.5" OR "GPT-4" OR "transformer language models")

AND

("rheumatology" OR "rheumatic disease" OR "arthritis" OR "rheumatoid arthritis" OR "osteoarthritis" OR "psoriatic arthritis" OR "ankylosing spondylitis" OR "lupus" OR "systemic lupus erythematosus" OR "scleroderma" OR "sjogren's syndrome" OR "gout" OR "vasculitis" OR "juvenile idiopathic arthritis" OR "fibromyalgia" OR "polymyalgia rheumatica" OR "dermatomyositis" OR "polymyositis" OR "mixed connective tissue disease" OR "behcet's disease")

**Scopus**

( ( TITLE-ABS ( "Natural Language Processing" OR "NLP" OR "Language Model" OR "Large Language Model" OR "LLM" OR "LLMs" OR "Generative Pre-trained Transformer" OR "GPT" OR "GPT-4" OR "GPT-3.5" OR "GPT-3" OR "ChatGPT" OR "BERT" OR "XLNet" OR "RoBERTa" OR "ELECTRA" OR "ALBERT" OR "Megatron" OR "Grok" OR "Galactica" OR "Gemini" OR "Claude" OR "LLaMA" OR "PaLM" ) ) AND ( TITLE-ABS ( "Rheumatology" OR "Rheumatic Disease" OR "Arthritis" OR "Rheumatoid Arthritis" OR "Osteoarthritis" OR "Psoriatic Arthritis" OR "Ankylosing Spondylitis" OR "Lupus" OR "Systemic Lupus Erythematosus" OR "Scleroderma" OR "Sjogren&apos;s Syndrome" OR "Gout" OR "Vasculitis" OR "Juvenile Idiopathic Arthritis" OR "Fibromyalgia" OR "Polymyalgia Rheumatica" OR "Dermatomyositis" OR "Polymyositis" OR "Mixed Connective Tissue Disease" OR "Behcet&apos;s Disease" ) ) ) AND PUBYEAR > 2001 AND PUBYEAR < 2025 AND ( LIMIT-TO ( SUBJAREA , "MEDI" ) ) AND ( LIMIT-TO ( DOCTYPE , "ar" ) ) AND ( LIMIT-TO ( EXACTKEYWORD , "Human" ) ) AND ( LIMIT-TO ( LANGUAGE , "English" ) )

**Cochrane Library**

("natural language processing" OR "NLP" OR "large language models" OR "LLM" OR "BERT" OR "GPT-3" OR "GPT" OR "ChatGPT" OR "GPT-3.5" OR "GPT-4" OR "transformer language models")

AND

("rheumatology" OR "rheumatic disease" OR "arthritis" OR "rheumatoid arthritis" OR "osteoarthritis" OR "psoriatic arthritis" OR "ankylosing spondylitis" OR "lupus" OR "systemic lupus erythematosus" OR "scleroderma" OR "sjogren's syndrome" OR "gout" OR "vasculitis" OR "juvenile idiopathic arthritis" OR "fibromyalgia" OR "polymyalgia rheumatica" OR "dermatomyositis" OR "polymyositis" OR "mixed connective tissue disease" OR "behcet's disease")

**Supplementary Table S1: Risk of bias assessment using the Quality Assessment Tool for Observational Cohort and Cross-Sectional Studies.**

| **Study (Ref)** | **Q1** | **Q2** | **Q3** | **Q4** | **Q5** | **Q6** | **Q7** | **Q8** | **Q9** | **Q10** | **Q11** | **overall** |
| --- | --- | --- | --- | --- | --- | --- | --- | --- | --- | --- | --- | --- |
| Chen et al. | Yes | Yes | Yes | Yes | Yes | Yes | Yes | Yes | Yes | Yes | Yes | Good |
| Saini et al. | Yes | Yes | Yes | No | Yes | NA | NA | Yes | Yes | No | No | Fair |
| Benavent et al. | Yes | Yes | Yes | No | Yes | Yes | NA | Yes | Yes | No | No | Fair |
| Li et al. (2022) | Yes | Yes | Yes | No | Yes | Yes | NA | Yes | Yes | NA | Yes | Good |
| Krusche et al. | Yes | Yes | Yes | No | No | NA | NA | Yes | Yes | Yes | No | Poor |
| Madrid‑García et al. | Yes | No | NA | No | NA | NA | No | Yes | Yes | Yes | No | Poor |
| Irfan et al. | Yes | Yes | NA | No | NA | NA | Yes | Yes | Yes | Yes | No | Fair |
| Nelson et al. | Yes | Yes | Yes | No | Yes | Yes | Yes | Yes | Yes | Yes | Yes | Good |
| Liu et al. | Yes | Yes | Yes | No | Yes | NA | Yes | Yes | Yes | Yes | Yes | Good |
| Zheng et al. | Yes | Yes | Yes | No | Yes | NA | NA | Yes | Yes | No | No | Fair |
| Humbert-Droz et al. | Yes | Yes | Yes | No | No | NA | Yes | Yes | Yes | No | Yes | Good |
| Benavent et al. | Yes | Yes | Yes | No | Yes | Yes | NA | Yes | Yes | No | No | Fair |
| VanSchaik et al. | Yes | Yes | Yes | Yes | Yes | NA | NA | Yes | Yes | Yes | Yes | Good |
| Walsh et al. | Yes | Yes | Yes | Yes | Yes | NA | NA | Yes | Yes | Yes | Yes | Good |
| Yoshida et al. | Yes | Yes | Yes | No | Yes | NA | NA | Yes | Yes | No | No | Fair |
| Li et al. (2023) | Yes | No | NA | No | NA | NA | No | Yes | Yes | Yes | No | Poor |
| Ye et al. | Yes | No | No | No | Yes | NA | Yes | Yes | Yes | Yes | No | Poor |
| Coskun et al. | Yes | No | NA | No | NA | NA | No | Yes | Yes | Yes | No | Poor |
| Liao et al. | Yes | Yes | Yes | No | Yes | Yes | NA | Yes | Yes | No | NA | Good |
| Lin et al. | Yes | Yes | Yes | Yes | Yes | NA | NA | Yes | Yes | Yes | No | Fair |
| Wang et al. | Yes | Yes | Yes | Yes | Yes | NA | NA | Yes | Yes | Yes | Yes | Good |
| Uz et al. | Yes | No | NA | No | NA | NA | Yes | Yes | Yes | Yes | Yes | Fair |
| Luedders et al. | Yes | Yes | Yes | No | Yes | Yes | NA | Yes | Yes | No | NA | Good |
| Osborne et al. | Yes | Yes | Yes | No | Yes | Yes | NA | Yes | Yes | No | NA | Good |
| Yang et al. | Yes | Yes | Yes | No | Yes | NA | Yes | Yes | Yes | Yes | Yes | Good |
| England et al. | Yes | Yes | Yes | No | Yes | Yes | NA | Yes | Yes | No | Yes | Good |
| Love et al. | Yes | Yes | Yes | No | Yes | Yes | Yes | Yes | Yes | NA | Yes | Good |
| Deng et al. | Yes | Yes | Yes | Yes | Yes | NA | NA | Yes | Yes | Yes | Yes | Good |
| van Leeuwen et al. | Yes | Yes | Yes | No | Yes | Yes | NA | Yes | Yes | NA | Yes | Good |
| Ivorra et al. | Yes | Yes | Yes | Yes | Yes | Yes | Yes | Yes | Yes | Yes | Yes | Good |
| Zhao et al. | Yes | Yes | Yes | No | Yes | NA | NA | Yes | Yes | NA | No | Fair |
| Kerr et al. | Yes | Yes | Yes | Yes | Yes | Yes | Yes | Yes | Yes | Yes | Yes | Good |
| Redd et al. | Yes | Yes | Yes | Yes | Yes | NA | NA | Yes | Yes | Yes | Yes | Good |
| Oliveira et al. | Yes | Yes | Yes | Yes | Yes | Yes | Yes | Yes | Yes | Yes | Yes | Good |
| Gräf et al. | No | Yes | No | No | NA | NA | No | Yes | Yes | Yes | No | Poor |

**Abbreviations**:Q1: Research Question Clearly Stated | Q2: Study Population Clearly Specified and Defined | Q3: Subjects Selected from Similar Populations with Uniform Criteria | Q4: Sample Size Justification and Power Description | Q5: Exposure Measured Prior to Outcome | Q6: Sufficient Timeframe to See an Association | Q7: Examination of Different Levels of Exposure | Q8: Clear, Valid, Reliable Exposure Measures | Q9: Outcome Measures Clearly Defined, Valid, Reliable | Q10: Blinding of Outcome Assessors to Exposure Status | Q11: Adjustment for Key Potential Confounding Variable.

**Supplementary Table S2: Summary of the conclusions, implications and limitations of the included studies.**

| Author, Year | Conclusions and Implications | Limitations |
| --- | --- | --- |
| Chen et al., 2023 | NLP techniques like synonym-based pain-level detection can effectively categorize pain severity in OA patients. | Uncertainty in determining if pain is related to OA or another issue. |
| Saini et al., 2023 | Ensemble models using NLP can automate severity prediction and report generation for knee OA, providing reliable clinical support. | Limited dataset; potential biases from in-house dataset characteristics; performance on diverse datasets untested. |
| Benavent et al., 2024 | NLP-based systems reliably extract clinical entities related to SpA, enhancing patient management through accurate data extraction. | Single-center study; need for external validation; limited ability to stratify subgroups. |
| Li et al., 2022 | Advanced NLP models like BERT significantly improve named entity recognition in clinical notes for RA, aiding in data extraction tasks. | Quality and consistency of manually annotated clinical notes; generalizability not evaluated. |
| Krusche et al., 2024 | NLP models like ChatGPT-4 can achieve comparable diagnostic accuracy to rheumatologists, suggesting potential use as a diagnostic support tool. | Small sample size; limited information; further studies needed on AI and symptom-checker applications. |
| Madrid-García et al., 2023 | GPT-4 demonstrates high accuracy and clinical reasoning in answering rheumatology questions, indicating its potential as an educational tool. | Potential for non-technical language use, unsuitable for certain applications. |
| Irfan et al., 2023 | GPT-4 provides comprehensive insights into Sjögren's Syndrome, highlighting its potential for detailed medical information extraction. | Potential oversimplification of information; citation inaccuracies; reliance on last training data up to 2021. |
| Nelson et al., 2015 | NLP improves the identification of infliximab infusions, enhancing accuracy in tracking treatment administration. | Potential inaccuracies in VHA coding practices; reliance solely on VHA data. |
| Liu et al., 2023 | Pretrained NLP models effectively extract medical entities and relationships from Chinese EMRs, supporting clinical data analysis. | Small number of CEMRs; complexity of Cypher query language. |
| Zheng et al., 2014 | NLP-ML methods accurately identify gout flares, outperforming traditional claims-based approaches. | Errors in identifying patients with multiple flares; lower specificity for >3 flares. |
| Humbert-Droz et al., 2023 | NLP pipelines can accurately extract RA outcome measures across diverse EHR systems, supporting large-scale clinical research. | Challenges in harmonizing raw notes; variations in note formatting; issues with nonnumerical entries. |
| Benavent et al., 2023 | EHRead® technology effectively identifies clinical variables for axSpA and PsA, demonstrating its utility in real-world settings. | Dependence on EHR accuracy; potential recall bias; data limited to three hospitals in Spain. |
| VanSchaik et al., 2023 | ELECTRA-based NLP models improve causal relationship extraction in biomedical literature, aiding in disease understanding. | Assumes one relationship per sentence; limited to explicit causal semantics. |
| Walsh et al., 2020 | NLP-assisted algorithms perform well in identifying axSpA patients, offering a robust tool for clinical research and management. | Limited generalizability; resource-intensive algorithms; not inclusive of ICD-10 codes. |
| Yoshida et al., 2024 | Combining NLP concepts with claims data enhances gout flare identification accuracy, providing a more comprehensive detection method. | Limited to single academic network; low mean capture proportion; potential mislabeling of gout flares. |
| Li et al., 2023 | Incorporating NLP models significantly improves question matching accuracy in RA question-answering systems. | Did not consider complex multi-round questioning; maintenance challenges as user problems become complex. |
| Ye et al., 2024 | Patients find AI-generated responses as comprehensive and readable as physician responses, though rheumatologists disagree. | Small sample size; limited to one academic center; predominantly older female patients. |
| Coskun et al., 2024 | GPT-4 excels in providing accurate and comprehensive information on methotrexate use, highlighting its educational potential. | Subjectivity in accuracy and completeness scores; limited to current versions of the models. |
| Liao et al., 2010 | HITEx system improves RA classification by integrating narrative and codified data, enhancing diagnostic accuracy. | Reliance on EMR data quality and comprehensiveness, which may vary across institutions. |
| Lin et al., 2015 | Automated systems using NLP accurately identify methotrexate-induced liver toxicity, supporting patient safety monitoring. | Lower PPV in Vanderbilt test set due to sparse temporal cues. |
| Wang et al., 2024 | NLP frameworks effectively discover and validate ADEs for DMARDs and biologics, bridging informatics and clinical practice. | ADEs may be natural consequences of RA; varying regimens among institutions. |
| Uz et al., 2023 | ChatGPT is a useful and reliable source for rheumatic disease information, though it may sometimes provide inconsistent answers. | ChatGPT may provide inconsistent answers to similar prompts. |
| Luedders et al., 2023 | NLP models enhance RA-ILD detection accuracy by extracting relevant terms from chest CT reports. | Potential bias in ILD-related term identification; reliance on radiologist documentation. |
| Osborne et al., 2024 | BERT-based algorithms effectively identify gout flares in ED patients, demonstrating strong predictive power. | Lack of comparison data; potential generalization issues. |
| Yang et al., 2024 | ChatGPT and Bard show varied concordance with AAOS CPGs, highlighting the need for further refinement of AI models. | Subjective classification of responses; near-perfect inter-rater reliability mitigates this. |
| England et al., 2024 | NLP increases the capture of accurate FVC values in RA-ILD, facilitating better disease monitoring. | Indirect assessment of NLP sensitivity likely underestimates true value. |
| Love et al., 2011 | NLP with EMR notes improves PsA classification accuracy, demonstrating its utility over coded data alone. | Difficulty in separating possible PsA cases from definite cases; dataset prevalence influences algorithm effectiveness. |
| Deng et al., 2022 | MetaMap mixed model improves lupus nephritis phenotype identification, enhancing clinical research capabilities. | Potential imperfections in gold standard labels; small external validation sample size. |
| van Leeuwen et al., 2024 | AI tools with NLP accurately identify AAV patients, outperforming traditional methods. | Potential for missed AAV patients; dependency on specific AI tools integrated with EHR systems. |
| Ivorra et al., 2024 | EHRead model accurately extracts clinical data for RA and ILD, supporting epidemiological research. | Incomplete or inconsistent EHR information; absence of structured data like radiology reports. |
| Zhao et al., 2020 | NLP-assisted algorithms accurately identify axSpA patients, offering robust tools for clinical research. | Variability in axSpA prevalence across settings; further validation needed in different EHR systems. |
| Kerr et al., 2015 | NLP systems effectively measure physician adherence to gout quality indicators, aiding in quality improvement efforts. | Variability in physician documentation; inability to validate undocumented BM counseling. |
| Redd et al., 2014 | NLP and SVM effectively detect SSc patients at risk for scleroderma renal crisis, supporting preventive measures. | Did not adjust for demographic variables; potential underreporting of localized scleroderma. |
| Oliveira et al., 2024 | RoBERTa and BioGPT models effectively detect gout flares, supporting early intervention in clinical settings. | Performance varied across datasets; lack of generalization. |
| Gräf et al., 2022 | Ada shows superior diagnostic accuracy for IRD compared to physicians, supporting its use as an early diagnostic tool. | Small sample size; potential user and disease dependency affecting diagnostic accuracy. |

**Abbreviations**: AI: Artificial Intelligence | ADE: Adverse Drug Events | AERS: Adverse Event Reporting System | AUC: Area Under the Curve | AUROC: Area Under the Receiver Operating Characteristic Curve | BM: Behavioral Modification | BERT: Bidirectional Encoder Representations from Transformers | CC: Chief Complaint | CEMRs: Chinese Electronic Medical Records | CPGs: Clinical Practice Guidelines | CT: Computed Tomography | DMARDs: Disease-Modifying Antirheumatic Drugs | EHR: Electronic Health Record | EMR: Electronic Medical Record | FVC: Forced Vital Capacity | GPT: Generative Pre-trained Transformer | HCPCS: Healthcare Common Procedure Coding System | HITEx: Health Information Text Extraction | ICD: International Classification of Diseases | ILD: Interstitial Lung Disease | IRD: Inflammatory Rheumatic Diseases | LLM: Large Language Model | NLP: Natural Language Processing | NER: Named Entity Recognition | NPV: Negative Predictive Value | OA: Osteoarthritis | PFT: Pulmonary Function Test | PPV: Positive Predictive Value | PsA: Psoriatic Arthritis | RA: Rheumatoid Arthritis | RA-ILD: Rheumatoid Arthritis-Associated Interstitial Lung Disease | ROC: Receiver Operating Characteristic | SVM: Support Vector Machine | SpA: Spondyloarthritis | STS: Semantic Text Similarity | VHA: Veterans Health Administration


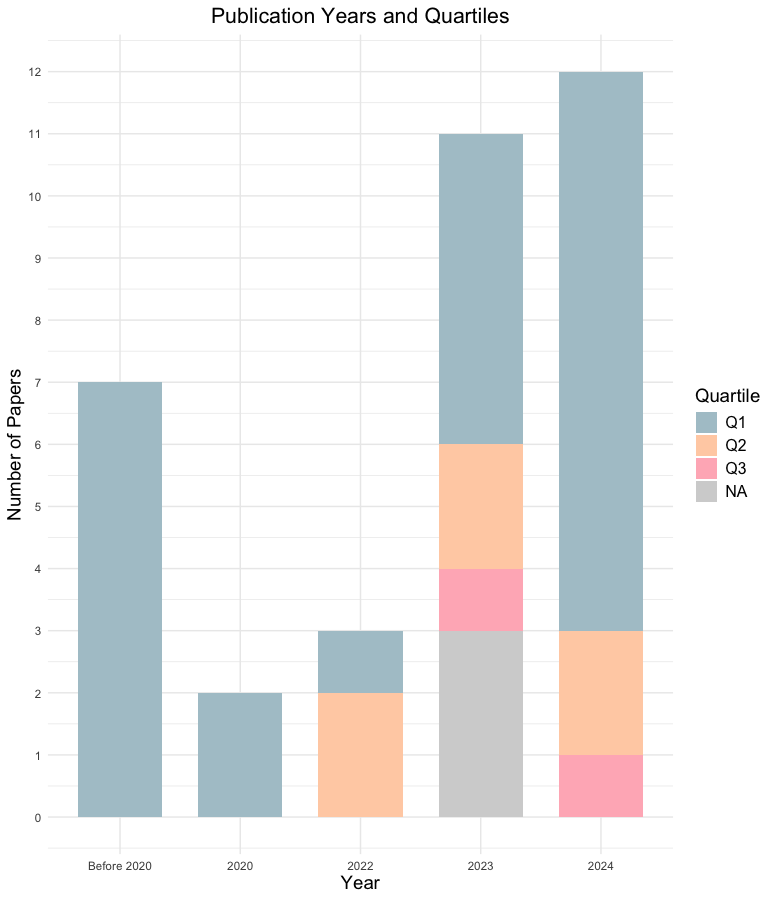


**Supplementary Figure S1:** Years of publication and quartiles of the included studies.
