# Supplementary figures and images for: Advancing rheumatology with natural language processing: insights and prospects from a systematic review
Source: Rheumatol Adv Pract. 2024 Sep 19;8(4):rkae120. doi: 10.1093/rap/rkae120 (PMC11467191; doi:10.1093/rap/rkae120)

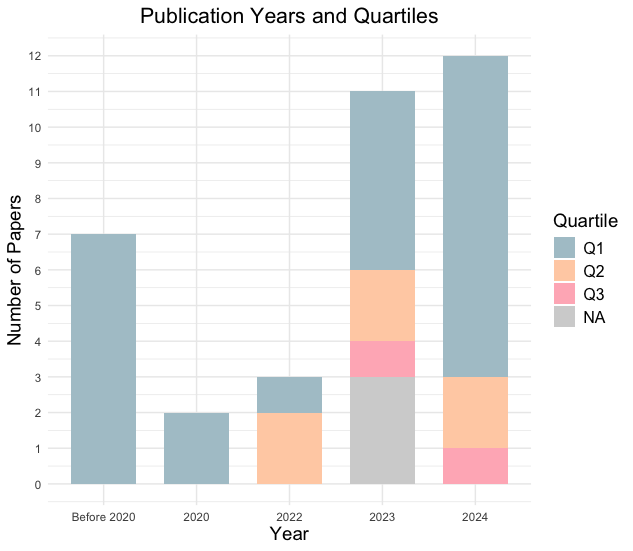

Supplement: rkae120_Supplementary_Data [file rkae120_supplementary_data.zip › RHEUMAP-2024-125.R1 - Sup Figure 1.TIFF]
